# Poly[[tri-μ-cyanido-cyanido(1,4,10,13-tetra­oxa-7,16-diaza­cyclo­octa­deca­ne)barium(II)platinum(II)] hemihydrate]

**DOI:** 10.1107/S1600536809008915

**Published:** 2009-03-19

**Authors:** Marilyn M. Olmstead, Christine M. Beavers, Latisha Paw U

**Affiliations:** a Department of Chemistry, University of California, Davis, CA 95616, USA

## Abstract

The title compound, {[BaPt(CN)_4_(C_12_H_26_N_2_O_4_)]·0.5H_2_O}_*n*_, is a two-dimensional coordination polymer in which the sheets are oriented approximately parallel to the (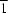
01) set of crystal planes. In the crystal structure, disordered water mol­ecules (half occupancy) connect the sheets into a three-dimensional network *via* inter­molecular O—H⋯O hydrogen bonds. An N—H⋯N inter­action is also present. The shortest Pt⋯Pt contacts are 7.5969 (4) Å by an inversion relationship and 7.6781 (4) Å by translation along the *a* axis.

## Related literature

For [BaPt(CN)_4_]·4H_2_O, see: Bergsoe *et al.* (1962 ▶); Williams *et al.* (1982 ▶). For the structure of a related salt, see: Olmstead *et al.* (2005 ▶).
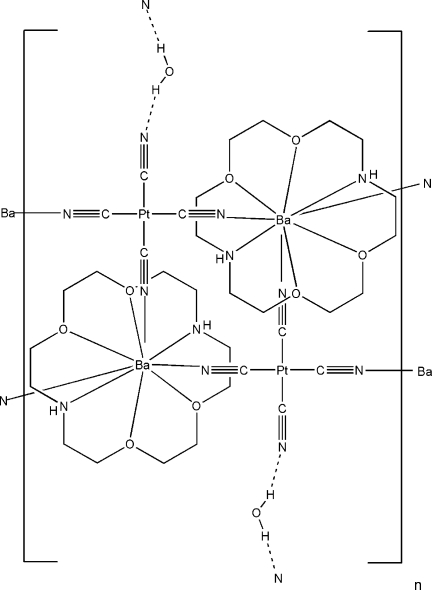


## Experimental

### 

#### Crystal data

[BaPt(CN)_4_(C_12_H_26_N_2_O_4_)]·0.5H_2_O
*M* *_r_* = 707.87
Monoclinic, 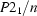

*a* = 7.6781 (4) Å
*b* = 14.8881 (9) Å
*c* = 20.2325 (12) Å
β = 93.254 (2)°
*V* = 2309.1 (2) Å^3^
*Z* = 4
Mo *K*α radiation
μ = 7.78 mm^−1^
*T* = 93 K
0.18 × 0.10 × 0.06 mm

#### Data collection

Bruker SMART APEXII diffractometer
Absorption correction: multi-scan (*SADABS*; Sheldrick, 1996 ▶) *T*_min_ = 0.469, *T*_max_ = 0.676 (expected range = 0.435–0.627)
30095 measured reflections
5292 independent reflections
5066 reflections with *I* > 2σ(*I*)
*R*_int_ = 0.027

#### Refinement

*R*[*F* ^2^ > 2σ(*F* ^2^)] = 0.013
*wR*(*F* ^2^) = 0.032
*S* = 1.03
5292 reflections
276 parameters
3 restraints
H atoms treated by a mixture of independent and constrained refinement
Δρ_max_ = 0.72 e Å^−3^
Δρ_min_ = −0.42 e Å^−3^

### 

Data collection: *APEX2* (Bruker, 2007 ▶); cell refinement: *SAINT* (Bruker, 2007 ▶); data reduction: *SAINT*; program(s) used to solve structure: *SHELXS97* (Sheldrick, 2008 ▶); program(s) used to refine structure: *SHELXL97* (Sheldrick, 2008 ▶); molecular graphics: *SHELXTL* (Sheldrick, 2008 ▶), *PovChem* (Thiessen, 2000 ▶) and *POV-RAY* (Cason *et al.*, 2004 ▶); software used to prepare material for publication: *SHELXL97*.

## Supplementary Material

Crystal structure: contains datablocks I, global. DOI: 10.1107/S1600536809008915/lh2782sup1.cif

Structure factors: contains datablocks I. DOI: 10.1107/S1600536809008915/lh2782Isup2.hkl

Additional supplementary materials:  crystallographic information; 3D view; checkCIF report

## Figures and Tables

**Table:** 

Ba1—O1	2.7831 (15)
Ba1—O2	2.8062 (15)
Ba1—O3	2.8261 (16)
Ba1—O4	2.7980 (15)
Ba1—N1^i^	2.814 (2)
Ba1—N3^ii^	2.8896 (19)
Ba1—N4	2.8431 (19)
Ba1—N5	2.8671 (18)
Ba1—N6	2.9291 (19)
Pt1—C1	1.981 (2)
Pt1—C2	2.003 (2)
Pt1—C3	1.995 (2)
Pt1—C4	1.985 (2)

**Table:** 

C1—Pt1—C2	87.61 (9)
C1—Pt1—C3	177.97 (10)
C1—Pt1—C4	89.54 (8)
C3—Pt1—C2	92.50 (8)
C4—Pt1—C2	176.47 (9)
C4—Pt1—C3	90.42 (8)

Symmetry codes: (i) 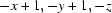; (ii) 
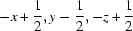
.

**Table 2:** Hydrogen-bond geometry (Å, °)

*D*—H⋯*A*	*D*—H	H⋯*A*	*D*⋯*A*	*D*—H⋯*A*
O5—H5*C*⋯N2	0.990 (10)	2.09 (4)	2.934 (5)	142 (5)
O5—H5*D*⋯N3^iv^	0.989 (10)	2.179 (14)	3.159 (4)	171 (5)
N6—H6⋯N1^i^	0.82 (3)	2.60 (3)	3.096 (3)	121 (2)

Symmetry codes: (i) 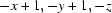; (iv) 
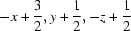
.

**Fig. 1.**
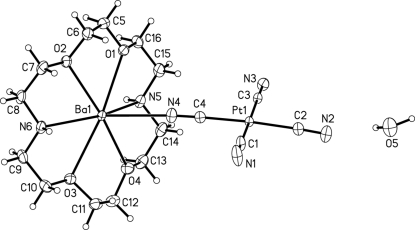
The asymmetric unit of the title compound with thermal ellipsoids shown at the 50% probability level. The water molecule of O5 is at half-occupancy.

**Fig. 2.**
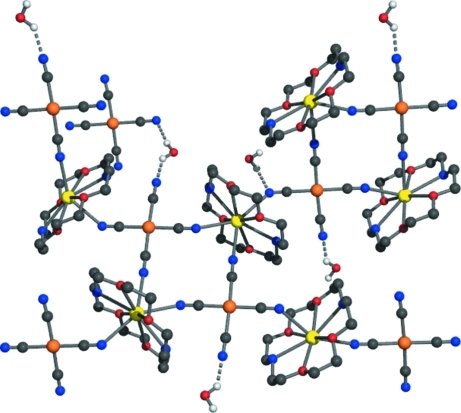
A view along the a axis direction showing the connection between cations, anions and water molecules. Color codes: Ba, yellow; Pt, orange; O, red; N, blue; C, gray; H, light gray. H-bonds are shown as dashed lines.

## supplementary crystallographic information

### Comment

Tetracyanoplatinate salts have a propensity toward the formation of columnar stacking motifs. Ba[Pt(CN)_4_], the accidental scintillation detector used by Roentgen (Bergsoe *et al.*, 1962), has this characteristic with a close Pt···Pt distance of 3.321 Å. The compound has optical and electrical properties that are orientation specific with respect to the crystallographic axes (Williams *et al.*, 1982). Even though the stacks of [Pt(CN)_4_]^2-^ are supported by bridging Ba^2+^*via* the N end of the cyano groups bonded to Pt, partial oxidation of these compounds, similar to that observed by a number of mono cationic salts, does not occur. Evidently, the mutual repulsion of the [Pt(CN)_4_]^2-^ groups cannot be overcome by the inability of the large Ba^2+^ coordination sphere to compress in a manner than matches the compression of the Pt chain, a change of *ca* 20% in the Pt···Pt separation. In this research, crown ethers were used to alter the coordination environment at Ba in order to assess the structural changes that would occur. A earlier report (Olmstead, *et al.*, 2005), focused on the salt [Ba(18-crown-6)(H_2_O)_2_][Pt(CN)_4_], 2.

The asymmetric unit of the title compound, [Ba(diaza-18-crown-6)][Pt(CN)_4_]^.^0.5 H_2_O, (1), is shown in Figure 1. Table 1 summarizes the coordination geometry for both Ba and Pt. The Ba^2+^ is coordinated by the diaza-18-crown-6 with average Ba—O distances of 2.80[2] Å and Ba—N distances of 2.89[4] Å (average deviations from the mean are given in square brackets). The Ba^2+^ is 0.39 (2) Å out of the N2O4 plane of the crown ether, giving the crown two distinct faces, an *endo* face and an *exo* face. The two aza-hydrogen atoms of the crown are in a *trans*- configuration. The Pt center shows normal square planar coordination geometry. The structure features a coordination polymer (Figure 2) in which barium achieves a total coordination number of nine. Three of the nitrogen ends of the cyano groups of [Pt(CN)_4_]^2-^, N1, N3, and N4, are coordinated to Ba1. The fourth cyano group is only involved in hydrogen bonding to the hemihydrate molecule which also participates in a hydrogen bond to N3. The C3—N3—Ba1 angle is more acute, at 135.06 (16)°, than either C1—N1—Ba1 (158.2 (2)°) or C4—N4—Ba1 (174.69 (17)°. One of the Ba—N bonds (N3) occurs on the *exo* face of the barium, and two (N1 and N4) occur on the *endo* face. These three bonds have an average length of 2.85[4] Å. The structure of (1) differs from the previously determined structure, (2), of [Ba(18-crown-6)(H_2_O)_2_][Pt(CN)_4_] (Olmstead *et al.*, 2005). In (2), Ba^2+^ has a coordination number of 10 and the donor set is comprised of six crown ether O, two water O and one cyano N on the *exo* side and one cyano N on the *endo* side. In (1), there is no water coordination to Ba. Instead, the water molecule is used in the creation of chains. Another interesting feature of (1) is the occurrence of a 12-atom (Ba—N—C—Pt—C—N)_2_ square ring motif about a center of inversion, as depicted in Figure 2. This motif does not appear in (2), which is rather more of a criss-cross structure.

There are no short Pt···Pt interactions in (1); the closest is at 7.5969 (4) Å by an inversion relationship, and the next closest is at 7.6781 (4) Å, by translation along the *a* axis.

### Experimental

A 61 mg portion of Ba[Pt(CN)_4_]^.^4H_2_O (0.12 mmol) and 37 mg (0.14 mmol) of diaza-18-crown-6 were dissolved in methanol. The solution was heated until the compounds dissolved, then cooled until a powder formed. The powder was collected and recrystallized in a minimum of methanol. After recrystallization, the crystals were once again dissolved in warm methanol. This solution was dispensed into 5 mm o.d. tubes, and layered with either water or ethanol. Crystals of the title compound formed after about 24 h. The crystal selected came from the ethanol-layered tube.

### Refinement

The occupancy of the water molecule was originally refined and converged at an occupancy of 0.41 (2). It was subsequently fixed at 0.50 occupancy. H atoms on water molecules were located in a difference Fourier map and refined with a distance constraint of 0.98 (1) Å for the O—H distance and 1.57 (3) Å for the H···H distance. Thermal parameters of the water H atoms were tied to 1.5 times that of the *U*_eq_(O). H atoms on aza groups were freely refined. All other H atoms were treated as riding on their parent C atoms, with C—H distances of 0.99 Å and *U*_iso_(H) = 1.2Ueq(C).

### Crystal data

**Table:**

[BaPt(CN)_4_(C_12_H_26_N_2_O_4_)]·0.5H_2_O	*F*(000) = 1340
*M**_r_* = 707.87	*D*_x_ = 2.036 Mg m^−^^3^
Monoclinic, *P*2_1_/*n*	Mo *K*α radiation, λ = 0.71073 Å
Hall symbol: -P 2yn	Cell parameters from 7683 reflections
*a* = 7.6781 (4) Å	θ = 2.4–33.6°
*b* = 14.8881 (9) Å	µ = 7.78 mm^−^^1^
*c* = 20.2325 (12) Å	*T* = 93 K
β = 93.254 (2)°	Prism, colorless
*V* = 2309.1 (2) Å^3^	0.18 × 0.10 × 0.06 mm
*Z* = 4	

### Data collection

**Table:**

Bruker SMART APEXII diffractometer	5292 independent reflections
Radiation source: fine-focus sealed tube	5066 reflections with *I* > 2σ(*I*)
graphite	*R*_int_ = 0.027
Detector resolution: 8.3 pixels mm^-1^	θ_max_ = 27.5°, θ_min_ = 1.7°
ω scans	*h* = −9→9
Absorption correction: multi-scan (*SADABS*; Sheldrick, 1996)	*k* = −19→19
*T*_min_ = 0.469, *T*_max_ = 0.676	*l* = −26→26
30095 measured reflections	

### Refinement

**Table:**

Refinement on *F*^2^	Primary atom site location: structure-invariant direct methods
Least-squares matrix: full	Secondary atom site location: difference Fourier map
*R*[*F*^2^ > 2σ(*F*^2^)] = 0.013	Hydrogen site location: inferred from neighbouring sites
*wR*(*F*^2^) = 0.032	H atoms treated by a mixture of independent and constrained refinement
*S* = 1.03	*w* = 1/[σ^2^(*F*_o_^2^) + (0.0143*P*)^2^ + 1.2267*P*] where *P* = (*F*_o_^2^ + 2*F*_c_^2^)/3
5292 reflections	(Δ/σ)_max_ = 0.003
276 parameters	Δρ_max_ = 0.72 e Å^−^^3^
3 restraints	Δρ_min_ = −0.42 e Å^−^^3^

### Special details

**Table:**

Geometry. All e.s.d.'s (except the e.s.d. in the dihedral angle between two l.s. planes) are estimated using the full covariance matrix. The cell e.s.d.'s are taken into account individually in the estimation of e.s.d.'s in distances, angles and torsion angles; correlations between e.s.d.'s in cell parameters are only used when they are defined by crystal symmetry. An approximate (isotropic) treatment of cell e.s.d.'s is used for estimating e.s.d.'s involving l.s. planes.
Refinement. Refinement of *F*^2^ against ALL reflections. The weighted *R*-factor *wR* and goodness of fit *S* are based on *F*^2^, conventional *R*-factors *R* are based on *F*, with *F* set to zero for negative *F*^2^. The threshold expression of *F*^2^ > σ(*F*^2^) is used only for calculating *R*-factors(gt) *etc*. and is not relevant to the choice of reflections for refinement. *R*-factors based on *F*^2^ are statistically about twice as large as those based on *F*, and *R*- factors based on ALL data will be even larger.

### Fractional atomic coordinates and isotropic or equivalent isotropic displacement parameters (Å^2^)

**Table:**

	*x*	*y*	*z*	*U*_iso_*/*U*_eq_	Occ. (<1)
Ba1	0.208165 (16)	0.303438 (8)	0.122058 (6)	0.01394 (3)	
Pt1	0.519371 (10)	0.670691 (5)	0.139759 (4)	0.01325 (3)	
O1	0.4491 (2)	0.29360 (10)	0.22989 (7)	0.0214 (3)	
O2	0.4371 (2)	0.15673 (10)	0.13466 (8)	0.0215 (3)	
O3	−0.0794 (2)	0.28946 (11)	0.02904 (8)	0.0238 (3)	
O4	−0.0661 (2)	0.42719 (11)	0.12179 (8)	0.0243 (3)	
O5	0.9018 (6)	1.0164 (3)	0.1191 (2)	0.0460 (10)	0.50
H5C	0.809 (6)	0.985 (4)	0.142 (3)	0.069*	0.50
H5D	0.944 (8)	1.063 (3)	0.151 (2)	0.069*	0.50
N1	0.6016 (3)	0.66373 (15)	−0.00967 (11)	0.0382 (6)	
N2	0.6503 (3)	0.87107 (13)	0.13625 (10)	0.0296 (5)	
N3	0.4574 (3)	0.67833 (12)	0.29213 (9)	0.0225 (4)	
N4	0.3748 (3)	0.47391 (13)	0.13041 (9)	0.0249 (4)	
N5	0.1327 (3)	0.39373 (13)	0.24229 (9)	0.0208 (4)	
H5	0.067 (4)	0.361 (2)	0.2607 (14)	0.033 (8)*	
N6	0.1924 (3)	0.15917 (12)	0.02347 (10)	0.0215 (4)	
H6	0.251 (4)	0.1801 (17)	−0.0055 (15)	0.031 (8)*	
C1	0.5688 (3)	0.66477 (15)	0.04481 (12)	0.0240 (5)	
C2	0.6033 (3)	0.79805 (15)	0.13932 (10)	0.0200 (4)	
C3	0.4780 (3)	0.67493 (13)	0.23617 (11)	0.0170 (4)	
C4	0.4298 (3)	0.54565 (14)	0.13458 (10)	0.0175 (4)	
C5	0.5553 (3)	0.21485 (16)	0.23661 (12)	0.0287 (5)	
H5A	0.4929	0.1678	0.2605	0.034*	
H5B	0.6649	0.2291	0.2626	0.034*	
C6	0.5967 (3)	0.18077 (16)	0.16941 (13)	0.0265 (5)	
H6A	0.6568	0.2280	0.1448	0.032*	
H6B	0.6745	0.1278	0.1739	0.032*	
C7	0.4592 (3)	0.09987 (15)	0.07817 (11)	0.0251 (5)	
H7A	0.5213	0.0441	0.0921	0.030*	
H7B	0.5289	0.1313	0.0456	0.030*	
C8	0.2811 (3)	0.07755 (15)	0.04759 (11)	0.0262 (5)	
H8A	0.2926	0.0352	0.0104	0.031*	
H8B	0.2109	0.0480	0.0809	0.031*	
C9	0.0142 (3)	0.14452 (17)	−0.00443 (13)	0.0305 (5)	
H9A	−0.0576	0.1176	0.0295	0.037*	
H9B	0.0161	0.1023	−0.0422	0.037*	
C10	−0.0645 (3)	0.23237 (17)	−0.02739 (12)	0.0305 (5)	
H10A	0.0104	0.2612	−0.0595	0.037*	
H10B	−0.1811	0.2220	−0.0495	0.037*	
C11	−0.1347 (3)	0.37872 (16)	0.01136 (12)	0.0268 (5)	
H11A	−0.2267	0.3764	−0.0249	0.032*	
H11B	−0.0351	0.4136	−0.0041	0.032*	
C12	−0.2041 (3)	0.42288 (17)	0.07133 (12)	0.0284 (5)	
H12A	−0.2463	0.4841	0.0600	0.034*	
H12B	−0.3029	0.3877	0.0872	0.034*	
C13	−0.1241 (3)	0.46060 (16)	0.18350 (12)	0.0271 (5)	
H13A	−0.2026	0.4164	0.2030	0.033*	
H13B	−0.1893	0.5174	0.1760	0.033*	
C14	0.0335 (3)	0.47660 (15)	0.23000 (11)	0.0257 (5)	
H14A	0.1096	0.5221	0.2106	0.031*	
H14B	−0.0047	0.5004	0.2725	0.031*	
C15	0.2917 (3)	0.40898 (16)	0.28449 (11)	0.0261 (5)	
H15A	0.2594	0.4311	0.3283	0.031*	
H15B	0.3630	0.4559	0.2643	0.031*	
C16	0.3982 (3)	0.32446 (15)	0.29356 (11)	0.0258 (5)	
H16A	0.5034	0.3366	0.3228	0.031*	
H16B	0.3286	0.2775	0.3146	0.031*	

### Atomic displacement parameters (Å^2^)

**Table:**

	*U*^11^	*U*^22^	*U*^33^	*U*^12^	*U*^13^	*U*^23^
Ba1	0.01958 (6)	0.01186 (6)	0.01060 (6)	−0.00094 (4)	0.00280 (4)	0.00008 (4)
Pt1	0.01580 (4)	0.01269 (4)	0.01157 (4)	−0.00147 (3)	0.00343 (3)	−0.00084 (3)
O1	0.0295 (9)	0.0185 (8)	0.0160 (7)	0.0012 (6)	−0.0015 (6)	−0.0014 (6)
O2	0.0250 (8)	0.0182 (8)	0.0213 (8)	0.0018 (6)	0.0009 (6)	−0.0040 (6)
O3	0.0294 (9)	0.0220 (8)	0.0196 (8)	0.0003 (7)	−0.0030 (7)	0.0013 (6)
O4	0.0240 (8)	0.0266 (9)	0.0224 (8)	0.0054 (7)	0.0017 (6)	−0.0021 (7)
O5	0.050 (2)	0.040 (2)	0.048 (2)	−0.0111 (19)	0.008 (2)	−0.0052 (19)
N1	0.0621 (16)	0.0335 (12)	0.0210 (11)	−0.0269 (11)	0.0185 (11)	−0.0095 (9)
N2	0.0400 (12)	0.0207 (10)	0.0293 (11)	−0.0052 (9)	0.0132 (9)	−0.0031 (8)
N3	0.0286 (11)	0.0216 (10)	0.0176 (10)	0.0007 (8)	0.0046 (8)	−0.0011 (7)
N4	0.0339 (11)	0.0202 (10)	0.0215 (10)	−0.0049 (8)	0.0087 (8)	−0.0026 (8)
N5	0.0300 (10)	0.0161 (9)	0.0170 (9)	0.0001 (8)	0.0068 (8)	−0.0001 (7)
N6	0.0315 (11)	0.0162 (9)	0.0171 (9)	−0.0034 (8)	0.0029 (8)	−0.0001 (7)
C1	0.0327 (13)	0.0184 (11)	0.0215 (12)	−0.0098 (9)	0.0072 (10)	−0.0035 (8)
C2	0.0231 (11)	0.0212 (11)	0.0162 (10)	−0.0025 (9)	0.0067 (8)	−0.0024 (8)
C3	0.0190 (10)	0.0131 (10)	0.0189 (11)	−0.0016 (8)	0.0016 (8)	−0.0003 (8)
C4	0.0202 (10)	0.0205 (11)	0.0124 (9)	−0.0008 (8)	0.0057 (8)	−0.0016 (8)
C5	0.0322 (13)	0.0233 (12)	0.0295 (13)	0.0052 (10)	−0.0076 (10)	−0.0015 (10)
C6	0.0243 (12)	0.0212 (12)	0.0338 (14)	0.0004 (9)	−0.0005 (10)	−0.0029 (10)
C7	0.0347 (13)	0.0181 (11)	0.0231 (11)	0.0055 (9)	0.0069 (10)	−0.0046 (9)
C8	0.0403 (14)	0.0146 (11)	0.0234 (11)	0.0002 (10)	0.0014 (10)	−0.0055 (9)
C9	0.0391 (14)	0.0240 (12)	0.0275 (13)	−0.0070 (11)	−0.0062 (11)	−0.0067 (10)
C10	0.0369 (14)	0.0306 (13)	0.0229 (12)	−0.0044 (11)	−0.0086 (10)	−0.0040 (10)
C11	0.0267 (12)	0.0284 (13)	0.0251 (12)	0.0018 (10)	−0.0015 (9)	0.0053 (10)
C12	0.0268 (12)	0.0293 (13)	0.0289 (12)	0.0051 (10)	0.0009 (10)	0.0056 (10)
C13	0.0304 (12)	0.0226 (12)	0.0294 (12)	0.0074 (10)	0.0107 (10)	−0.0013 (10)
C14	0.0368 (13)	0.0188 (11)	0.0224 (11)	0.0043 (10)	0.0098 (10)	−0.0034 (9)
C15	0.0419 (14)	0.0229 (12)	0.0137 (10)	−0.0020 (10)	0.0021 (9)	−0.0047 (9)
C16	0.0376 (14)	0.0245 (12)	0.0148 (11)	−0.0031 (10)	−0.0035 (10)	−0.0031 (9)

### Geometric parameters (Å, °)

**Table:**

Ba1—O1	2.7831 (15)	N6—C9	1.466 (3)
Ba1—O2	2.8062 (15)	N6—H6	0.82 (3)
Ba1—O3	2.8261 (16)	C5—C6	1.502 (3)
Ba1—O4	2.7980 (15)	C5—H5A	0.9900
Ba1—N1^i^	2.814 (2)	C5—H5B	0.9900
Ba1—N3^ii^	2.8896 (19)	C6—H6A	0.9900
Ba1—N4	2.8431 (19)	C6—H6B	0.9900
Ba1—N5	2.8671 (18)	C7—C8	1.506 (3)
Ba1—N6	2.9291 (19)	C7—H7A	0.9900
Pt1—Pt1^i^	7.5969 (4)	C7—H7B	0.9900
Pt1—Pt1^iii^	7.6781 (4)	C8—H8A	0.9900
Pt1—C1	1.981 (2)	C8—H8B	0.9900
Pt1—C2	2.003 (2)	C9—C10	1.503 (4)
Pt1—C3	1.995 (2)	C9—H9A	0.9900
Pt1—C4	1.985 (2)	C9—H9B	0.9900
O1—C5	1.430 (3)	C10—H10A	0.9900
O1—C16	1.442 (3)	C10—H10B	0.9900
O2—C6	1.424 (3)	C11—C12	1.504 (3)
O2—C7	1.440 (3)	C11—H11A	0.9900
O3—C10	1.433 (3)	C11—H11B	0.9900
O3—C11	1.434 (3)	C12—H12A	0.9900
O4—C12	1.431 (3)	C12—H12B	0.9900
O4—C13	1.438 (3)	C13—C14	1.509 (3)
O5—H5C	0.990 (10)	C13—H13A	0.9900
O5—H5D	0.989 (10)	C13—H13B	0.9900
N1—C1	1.145 (3)	C14—H14A	0.9900
N2—C2	1.148 (3)	C14—H14B	0.9900
N3—C3	1.153 (3)	C15—C16	1.506 (3)
N4—C4	1.150 (3)	C15—H15A	0.9900
N5—C14	1.464 (3)	C15—H15B	0.9900
N5—C15	1.467 (3)	C16—H16A	0.9900
N5—H5	0.81 (3)	C16—H16B	0.9900
N6—C8	1.463 (3)		
			
O1—Ba1—O4	120.20 (4)	N4—C4—Pt1	178.3 (2)
O1—Ba1—O2	60.18 (4)	O1—C5—C6	109.86 (19)
O4—Ba1—O2	168.59 (5)	O1—C5—H5A	109.7
O1—Ba1—N1^i^	106.86 (6)	C6—C5—H5A	109.7
O4—Ba1—N1^i^	108.01 (6)	O1—C5—H5B	109.7
O2—Ba1—N1^i^	81.80 (6)	C6—C5—H5B	109.7
O1—Ba1—O3	167.83 (5)	H5A—C5—H5B	108.2
O4—Ba1—O3	59.19 (5)	O2—C6—C5	108.1 (2)
O2—Ba1—O3	117.70 (5)	O2—C6—H6A	110.1
N1^i^—Ba1—O3	84.06 (6)	C5—C6—H6A	110.1
O1—Ba1—N4	73.94 (5)	O2—C6—H6B	110.1
O4—Ba1—N4	75.43 (5)	C5—C6—H6B	110.1
O2—Ba1—N4	114.32 (5)	H6A—C6—H6B	108.4
N1^i^—Ba1—N4	68.93 (6)	O2—C7—C8	108.08 (18)
O3—Ba1—N4	115.93 (5)	O2—C7—H7A	110.1
O1—Ba1—N5	61.12 (5)	C8—C7—H7A	110.1
O4—Ba1—N5	60.30 (5)	O2—C7—H7B	110.1
O2—Ba1—N5	116.33 (5)	C8—C7—H7B	110.1
N1^i^—Ba1—N5	138.01 (6)	H7A—C7—H7B	108.4
O3—Ba1—N5	114.31 (5)	N6—C8—C7	110.40 (19)
N4—Ba1—N5	69.08 (5)	N6—C8—H8A	109.6
O1—Ba1—N3^ii^	77.91 (5)	C7—C8—H8A	109.6
O4—Ba1—N3^ii^	93.95 (5)	N6—C8—H8B	109.6
O2—Ba1—N3^ii^	74.83 (5)	C7—C8—H8B	109.6
N1^i^—Ba1—N3^ii^	149.73 (6)	H8A—C8—H8B	108.1
O3—Ba1—N3^ii^	89.94 (5)	N6—C9—C10	109.8 (2)
N4—Ba1—N3^ii^	138.73 (5)	N6—C9—H9A	109.7
N5—Ba1—N3^ii^	71.06 (5)	C10—C9—H9A	109.7
O1—Ba1—N6	119.68 (5)	N6—C9—H9B	109.7
O4—Ba1—N6	118.65 (5)	C10—C9—H9B	109.7
O2—Ba1—N6	59.51 (5)	H9A—C9—H9B	108.2
N1^i^—Ba1—N6	65.19 (6)	O3—C10—C9	108.59 (19)
O3—Ba1—N6	59.47 (5)	O3—C10—H10A	110.0
N4—Ba1—N6	134.12 (5)	C9—C10—H10A	110.0
N5—Ba1—N6	156.79 (6)	O3—C10—H10B	110.0
N3^ii^—Ba1—N6	86.21 (5)	C9—C10—H10B	110.0
C1—Pt1—C2	87.61 (9)	H10A—C10—H10B	108.4
C1—Pt1—C3	177.97 (10)	O3—C11—C12	108.50 (19)
C1—Pt1—C4	89.54 (8)	O3—C11—H11A	110.0
C3—Pt1—C2	92.50 (8)	C12—C11—H11A	110.0
C4—Pt1—C2	176.47 (9)	O3—C11—H11B	110.0
C4—Pt1—C3	90.42 (8)	C12—C11—H11B	110.0
C5—O1—C16	110.98 (17)	H11A—C11—H11B	108.4
C5—O1—Ba1	117.88 (12)	O4—C12—C11	108.26 (19)
C16—O1—Ba1	118.79 (13)	O4—C12—H12A	110.0
C6—O2—C7	113.74 (17)	C11—C12—H12A	110.0
C6—O2—Ba1	111.50 (12)	O4—C12—H12B	110.0
C7—O2—Ba1	119.14 (13)	C11—C12—H12B	110.0
C10—O3—C11	112.75 (17)	H12A—C12—H12B	108.4
C10—O3—Ba1	118.66 (13)	O4—C13—C14	108.62 (18)
C11—O3—Ba1	107.88 (13)	O4—C13—H13A	110.0
C12—O4—C13	112.47 (17)	C14—C13—H13A	110.0
C12—O4—Ba1	119.92 (13)	O4—C13—H13B	110.0
C13—O4—Ba1	119.74 (13)	C14—C13—H13B	110.0
H5C—O5—H5D	104 (3)	H13A—C13—H13B	108.3
C1—N1—Ba1^i^	158.2 (2)	N5—C14—C13	111.34 (19)
C3—N3—Ba1^iv^	135.06 (16)	N5—C14—H14A	109.4
C4—N4—Ba1	174.69 (17)	C13—C14—H14A	109.4
C14—N5—C15	112.09 (18)	N5—C14—H14B	109.4
C14—N5—Ba1	112.29 (13)	C13—C14—H14B	109.4
C15—N5—Ba1	111.39 (13)	H14A—C14—H14B	108.0
C14—N5—H5	105 (2)	N5—C15—C16	111.70 (18)
C15—N5—H5	110 (2)	N5—C15—H15A	109.3
Ba1—N5—H5	106 (2)	C16—C15—H15A	109.3
C8—N6—C9	114.29 (18)	N5—C15—H15B	109.3
C8—N6—Ba1	112.42 (13)	C16—C15—H15B	109.3
C9—N6—Ba1	111.89 (14)	H15A—C15—H15B	107.9
C8—N6—H6	107 (2)	O1—C16—C15	109.27 (18)
C9—N6—H6	109 (2)	O1—C16—H16A	109.8
Ba1—N6—H6	101.8 (19)	C15—C16—H16A	109.8
N1—C1—Pt1	177.6 (2)	O1—C16—H16B	109.8
N2—C2—Pt1	177.15 (19)	C15—C16—H16B	109.8
N3—C3—Pt1	178.6 (2)	H16A—C16—H16B	108.3
			
O4—Ba1—O1—C5	162.79 (15)	N6—Ba1—O4—C13	142.47 (15)
O2—Ba1—O1—C5	−4.04 (14)	O1—Ba1—N5—C14	146.68 (17)
N1^i^—Ba1—O1—C5	−73.83 (16)	O4—Ba1—N5—C14	−20.73 (14)
O3—Ba1—O1—C5	79.3 (3)	O2—Ba1—N5—C14	171.63 (14)
N4—Ba1—O1—C5	−135.26 (16)	N1^i^—Ba1—N5—C14	63.44 (19)
N5—Ba1—O1—C5	150.13 (17)	O3—Ba1—N5—C14	−45.94 (16)
N3^ii^—Ba1—O1—C5	75.26 (15)	N4—Ba1—N5—C14	64.00 (15)
N6—Ba1—O1—C5	−3.21 (17)	N3^ii^—Ba1—N5—C14	−126.96 (16)
O4—Ba1—O1—C16	23.94 (15)	N6—Ba1—N5—C14	−114.77 (18)
O2—Ba1—O1—C16	−142.89 (15)	O1—Ba1—N5—C15	20.02 (13)
N1^i^—Ba1—O1—C16	147.32 (14)	O4—Ba1—N5—C15	−147.39 (16)
O3—Ba1—O1—C16	−59.6 (3)	O2—Ba1—N5—C15	44.97 (15)
N4—Ba1—O1—C16	85.89 (14)	N1^i^—Ba1—N5—C15	−63.22 (18)
N5—Ba1—O1—C16	11.28 (14)	O3—Ba1—N5—C15	−172.60 (13)
N3^ii^—Ba1—O1—C16	−63.59 (14)	N4—Ba1—N5—C15	−62.66 (14)
N6—Ba1—O1—C16	−142.06 (14)	N3^ii^—Ba1—N5—C15	106.38 (15)
O1—Ba1—O2—C6	−28.85 (14)	N6—Ba1—N5—C15	118.57 (17)
O4—Ba1—O2—C6	−124.0 (2)	O1—Ba1—N6—C8	16.47 (17)
N1^i^—Ba1—O2—C6	86.00 (15)	O4—Ba1—N6—C8	−149.75 (14)
O3—Ba1—O2—C6	164.83 (14)	O2—Ba1—N6—C8	17.30 (14)
N4—Ba1—O2—C6	23.63 (15)	N1^i^—Ba1—N6—C8	112.45 (17)
N5—Ba1—O2—C6	−54.05 (15)	O3—Ba1—N6—C8	−149.49 (17)
N3^ii^—Ba1—O2—C6	−113.42 (15)	N4—Ba1—N6—C8	112.74 (15)
N6—Ba1—O2—C6	151.98 (16)	N5—Ba1—N6—C8	−68.9 (2)
O1—Ba1—O2—C7	−164.49 (16)	N3^ii^—Ba1—N6—C8	−57.32 (15)
O4—Ba1—O2—C7	100.3 (3)	O1—Ba1—N6—C9	146.66 (14)
N1^i^—Ba1—O2—C7	−49.64 (15)	O4—Ba1—N6—C9	−19.55 (17)
O3—Ba1—O2—C7	29.19 (16)	O2—Ba1—N6—C9	147.49 (17)
N4—Ba1—O2—C7	−112.01 (15)	N1^i^—Ba1—N6—C9	−117.35 (17)
N5—Ba1—O2—C7	170.31 (14)	O3—Ba1—N6—C9	−19.29 (14)
N3^ii^—Ba1—O2—C7	110.94 (15)	N4—Ba1—N6—C9	−117.06 (16)
N6—Ba1—O2—C7	16.34 (14)	N5—Ba1—N6—C9	61.3 (2)
O1—Ba1—O3—C10	−104.1 (2)	N3^ii^—Ba1—N6—C9	72.88 (15)
O4—Ba1—O3—C10	165.23 (17)	C16—O1—C5—C6	176.36 (19)
O2—Ba1—O3—C10	−27.36 (16)	Ba1—O1—C5—C6	34.5 (2)
N1^i^—Ba1—O3—C10	50.13 (16)	C7—O2—C6—C5	−163.08 (18)
N4—Ba1—O3—C10	113.22 (15)	Ba1—O2—C6—C5	58.8 (2)
N5—Ba1—O3—C10	−169.25 (15)	O1—C5—C6—O2	−62.2 (2)
N3^ii^—Ba1—O3—C10	−100.14 (16)	C6—O2—C7—C8	178.29 (19)
N6—Ba1—O3—C10	−14.51 (15)	Ba1—O2—C7—C8	−47.0 (2)
O1—Ba1—O3—C11	126.2 (2)	C9—N6—C8—C7	−177.3 (2)
O4—Ba1—O3—C11	35.48 (12)	Ba1—N6—C8—C7	−48.3 (2)
O2—Ba1—O3—C11	−157.11 (12)	O2—C7—C8—N6	62.9 (2)
N1^i^—Ba1—O3—C11	−79.61 (13)	C8—N6—C9—C10	179.8 (2)
N4—Ba1—O3—C11	−16.52 (14)	Ba1—N6—C9—C10	50.6 (2)
N5—Ba1—O3—C11	61.00 (14)	C11—O3—C10—C9	173.3 (2)
N3^ii^—Ba1—O3—C11	130.11 (13)	Ba1—O3—C10—C9	45.8 (2)
N6—Ba1—O3—C11	−144.25 (14)	N6—C9—C10—O3	−64.2 (3)
O1—Ba1—O4—C12	−170.45 (14)	C10—O3—C11—C12	161.05 (19)
O2—Ba1—O4—C12	−81.9 (3)	Ba1—O3—C11—C12	−65.97 (19)
N1^i^—Ba1—O4—C12	66.73 (16)	C13—O4—C12—C11	−174.38 (19)
O3—Ba1—O4—C12	−4.57 (14)	Ba1—O4—C12—C11	−25.4 (2)
N4—Ba1—O4—C12	128.36 (16)	O3—C11—C12—O4	61.3 (2)
N5—Ba1—O4—C12	−157.69 (17)	C12—O4—C13—C14	−171.16 (19)
N3^ii^—Ba1—O4—C12	−92.14 (15)	Ba1—O4—C13—C14	39.8 (2)
N6—Ba1—O4—C12	−4.31 (17)	C15—N5—C14—C13	176.85 (18)
O1—Ba1—O4—C13	−23.67 (17)	Ba1—N5—C14—C13	50.6 (2)
O2—Ba1—O4—C13	64.9 (3)	O4—C13—C14—N5	−59.8 (2)
N1^i^—Ba1—O4—C13	−146.49 (15)	C14—N5—C15—C16	−176.63 (19)
O3—Ba1—O4—C13	142.21 (16)	Ba1—N5—C15—C16	−49.9 (2)
N4—Ba1—O4—C13	−84.86 (15)	C5—O1—C16—C15	178.20 (19)
N5—Ba1—O4—C13	−10.91 (15)	Ba1—O1—C16—C15	−40.3 (2)
N3^ii^—Ba1—O4—C13	54.64 (16)	N5—C15—C16—O1	60.5 (2)

Symmetry codes: (i) −*x*+1, −*y*+1, −*z*; (ii) −*x*+1/2, *y*−1/2, −*z*+1/2; (iii) *x*+1, *y*, *z*; (iv) −*x*+1/2, *y*+1/2, −*z*+1/2.

### Hydrogen-bond geometry (Å, °)

**Table:**

*D*—H···*A*	*D*—H	H···*A*	*D*···*A*	*D*—H···*A*
O5—H5C···N2	0.99 (1)	2.09 (4)	2.934 (5)	142 (5)
O5—H5D···N3^v^	0.99 (1)	2.18 (1)	3.159 (4)	171 (5)
N6—H6···N1^i^	0.82 (3)	2.60 (3)	3.096 (3)	121 (2)

Symmetry codes: (v) −*x*+3/2, *y*+1/2, −*z*+1/2; (i) −*x*+1, −*y*+1, −*z*.
